# Finding common (research) ground between general practitioners and neuroscientists: the vital role of knowledge circulation in closing the evidence-to-practice gap

**DOI:** 10.1186/s12875-021-01560-3

**Published:** 2021-10-20

**Authors:** Astrid Eich-Krohm, Bernt-Peter Robra, Yvonne Marx, Markus Herrmann

**Affiliations:** 1 Faculty of Medicine, Otto-von-Guericke-University Magdeburg, Institute of Social Medicine and Health Systems Research, Leipziger Straße 44, 39120 Magdeburg, Germany; 2 Faculty of Medicine, Otto-von-Guericke-University Magdeburg, Institute of General Practice, Leipziger Straße 44, 39120 Magdeburg, Germany

**Keywords:** Dementia, Knowledge translation, Knowledge circulation, General practitioners, Neuroscientists, Research paradigms

## Abstract

**Background:**

It may take 15 years or longer before research evidence is integrated into clinical practice. This evidence-to-practice gap has deleterious effects on patients as well as research and clinical processes. Bringing clinical knowledge into the research process, however, has the potential to close the evidence-to-practice gap. The NEUROTRANS-Project attempts to bring research and practice together by focusing on two groups that usually operate separately in their communities: general practitioners and neuroscientists. Although both groups focus on dementia as an area of work, they do so in different contexts and without opportunities to share their expertise. Finding new treatment pathways for patients with dementia will require an equal knowledge exchange among researchers and clinicians along with the integration of that knowledge into research processes, so that both groups will benefit from the expertise of the other.

**Methods:**

The NEUROTRANS-Project uses a qualitative, multi-stage research design to explore how neuroscientists and general practitioners (GPs) approach dementia. Using a grounded theory methodology, it analyzes semi-structured interviews, case vignettes, focus groups with GPs in Saxony-Anhalt, Germany, and informal conversations with, and observations of, neuroscientists from the German Center for Neurodegenerative Diseases in Magdeburg.

**Results:**

The NEUROTRANS-Project identified a clear division of labor between two highly specialized professional groups. Neuroscientists focus abstractly on nosology whereas general practitioners tend to patient care following a hermeneutic approach integrating the patients’ perspective of illness. These different approaches to dementia create a barrier to constructive dialogue and the capacity of these groups to do research together with a common aim. Additionally, the broader system of research funding and health care within which the two groups operate reinforces their divide thereby limiting joint research capacity.

**Conclusions:**

Overcoming barriers to research collaboration between general practitioners and neuroscientists requires a shift in perspective in which both groups actively engage with the other’s viewpoints to facilitate knowledge circulation (KC). Bringing ‘art into science and science into art’, i.e. amalgamating the hermeneutic approach with the perspective of nosology, is the first step in developing joint research agendas that have the potential to close the evidence-to-practice gap.

**Supplementary Information:**

The online version contains supplementary material available at 10.1186/s12875-021-01560-3.

## Background

Dementia is a complex range of symptoms including memory loss and difficulties of understanding. Because age is the number one risk factor for dementia the continuous increase in life-expectancy will affect more people by some form of it. While general practitioners (GPs) confront multifaceted care management for their patients with dementia and their illness-perspective, neuroscientists try to find the causes and develop medical treatment for the disease. Both groups gain important knowledge in their own right. However, it is unclear, how much knowledge exchange, or circulation, occurs between them. The NEUROTRANS-Project aimed to find out.

Different terms have been used to describe how research evidence reaches the clinical practitioner, most often “implementation” and “knowledge translation”. Implementation refers to strategies that support the transfer of research knowledge into practice by examining barriers and support systems [1]. Knowledge Translation (KT) as defined by of the Canadian Institute of Health Research is “a dynamic and iterative process that includes the synthesis, dissemination, exchange and ethically sound application of knowledge to improve health, provide more effective health services and products, and strengthen the health care system” [2]. Both systems recognize the practitioner but not as a researcher. However, we argue that research projects should be developed jointly by equally utilizing the knowledge of the practitioner as well. Therefore, knowledge circulation (KC) best captures the on-going exchange of knowledge between research and practice [1].

GPs are the first to see patients with cognitive, social or behavioral changes and other non-specific signs and based on those observations to decide which different clinical and integrated care pathways they have to choose for detection and management of dementia and other diseases [3–7]. In contrast, neuroscientists use research to understand causation and disease progression by focusing on observable changes in the brain. These different approaches to detect and diagnose dementia in patients are usually not shared because GPs and neuroscientists act isolated from each other. Therefore, the NEUROTRANS Project aimed to foster an equal exchange between practitioners and scientists grounded in the idea that both groups could benefit from the expertise of the other.

Even though dementia has been researched extensively for the last 40 years, science is far from identifying a valid method of timely detection or an effective treatment [8].. Existing research paradigms such as bench to bedside (B to B) and randomized controlled trials (RCTs) offer a one-way street toward progress: scientists collect and analyze data and, after many years of pre-clinical and clinical research, hope their findings will be implemented into medical practice. Despite the steady increase in ‘B to B projects’ and RCTs during the last 20 years, there remains an evidence-to-practice gap when it comes to the implementation of evidence-based interventions in primary care [9]. As a result, research outcomes either do not reach practitioners and their patients or they do so only after extensive delay [10, 11].

We argue that the evidence-to-practice gap is partially based on how the research paradigm is structured by ignoring the insights and expertise of practitioners in the field. Evidence Based Medicine (EBM) posit that practitioners combine the best available evidence with their clinical and practical experience within the context of each patient to determine the best course of action [12]. Ioannidis [11] suggests that to improve research findings communication should be multidisciplinary and start with designing the study. Whereas EBM and RCTs are disease-oriented practitioners are patient-oriented putting the social context of the patient first [13]. In order to bring both concepts closer together communication between researchers and practitioners needs stronger support from both sides [13].

Therefore, this study focuses on the communication between neuroscientists and general practitioners about patients with dementia. We use the concept of ‘art into science (patient centered focus) and science into art (research centered focus)’ to describe an interdisciplinary collaboration on equal terms between GPs and neuroscientists. In the German context, many neuroscientists are physicians and general physicians are involved in research as well. We do not want to suggest that both groups have no knowledge of the other. Knowledge exchanges should occur in both directions to strengthen research and patients while taking into account ethical, social, cultural, professional, and other normative factors as supported by the „Ethical Legal and Social Aspects “(ELSA) initiative of the German Ministry of Education and Research [14, 15].

The research questions were:

- How can GPs contribute to neuroscience research about dementia and
- How can neuroscientists support GPs with dementia patients?

## Methods

**Fig. 1:**
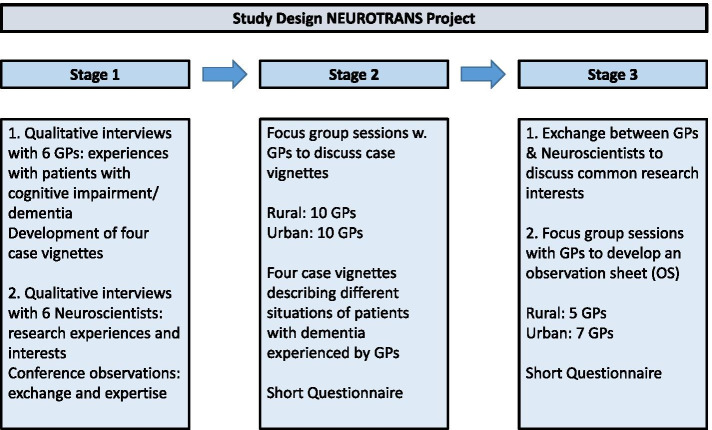
NEUROTRANS Multi-stage research design

Participatory research is a useful strategy to develop science that enables practitioners to contribute to and develop practice-based research. At the same time, it offers foundational researchers new views to ultimately benefit the patient [5]. The NEUROTRANS-Project uses an exploratory qualitative, multi-stage research design. We conducted semi-structured interviews, (out of which case vignettes were developed), focus groups, observations, and informal conversations (Fig. 1). Participation in the project was voluntary and approved by the Ethics committee of the university (see Declarations). We pseudonymized participating GPs and neuroscientists in the text. Figure 1 shows the different parts of the multi-stage research design. The research study was approved by the Ethics Committee of the Otto-von-Guericke-University Magdeburg (Number: 72/13, May 15, 2013). More information under “Declarations” page 22.

The theoretical sampling frame considered both groups. The German Center of Neurodegenerative Disease (Deutsches Zentrum für Neurodegenerative Erkrankungen = DZNE) in Magdeburg had already declared their interest to participate while the NEUROTRANS-project developed. For the interviews with the neuroscientists we contacted the leaders of the different working groups at the DZNE. Additionally, we collected informal observations of the neuroscientists and their wider research context at three conferences that took place at the DZNE during the time of the NEUROTRANS project. The research was approved by the Ethics Committee of the Otto-von-Guericke-University Magdeburg (Number: 72/13, May 15, 2013). This research was conducted in accordance with international guidelines and the ethical standards outlined in the Declaration of Helsinki (for more information please review the declarations on page 22).

The participating GPs were recruited through the active pool of GPs who collaborate with the university in the education of medical students. Twenty GPs in the area were contacted, six agreed to be interviewed. In contrast, the focus-group participants were recruited through so called Quality Circles (QCs) that offer continuing professional development (CPD). In Saxony-Anhalt many GPs participate in quality circles in which three to ten physicians meet regularly to discuss technical and organizational problems in search of respective solutions or improvements. The participation of GPs in quality circles is voluntary, but part of an obligatory CPD process. Each quality circle has a moderator who facilitates the group meeting and participants contribute to the groups’ progress from a physician’s perspective and for planning and quality control of office procedures [16]. Participating physicians also develop competencies they can only gain through regular peer-to-peer exchange. As a continuing professional development CPD method, quality circles reinforce cooperation in problem solving, self-learning, and transfer oriented training processes [1, 17, 18]. Saxony-Anhalt has a total of 203 quality circles with an average of nine members who meet five times per year for 90 to 120 min [19]. We asked the moderators of eight quality circles in one rural and one urban area of Saxony-Anhalt if their groups would like to participate in focus group sessions. The moderators then discussed the question with their participating GPs and two groups decided to participate. In the following we describe the three stages of the study.

### Stage 1: semi-structured interviews and observations

In order to bring GPs and neuroscientists together, we first sought to understand the groups separately. Thus, the first stage of the NEUROTRANS-Project included six semi-structured interviews with GPs to learn how they detect signs of dementia, develop strategies of diagnosis and disclosure to patients and relatives, and create treatment plans. The interviews were conducted in the offices of the GPs and lasted between 30 and 90 min (average 60 min). The questions asked were broad so that the GPs were able to tell a story, for example: “how do you recognize memory changes in your patients? Can you tell me different examples?” To take note of emerging themes, each interview was audio recorded, transcribed, coded, and analyzed using the constant comparative method [20]. The coordinating researcher is a medical sociologist and a registered nurse trained in qualitative methods and has extensive experience in conducting and analyzing semi-structured interviews as well as focus group moderation. The software program MAXQDA supported the management of the data.

The standard procedure of Grounded Theory was applied to systematically and continuously develop the theoretical codes, their properties and dimensions so that the full range of codes and their interconnectedness became visible [20, 21]. The coding process started with open coding (for example “recognizing memory changes in patients”), followed by axial coding, for example behavioral changes of patients categorized into health outcomes (missing doctor’s appointments) or personal behavior (wearing stained clothes) and ending with selective coding to develop core categories. This process was also helpful in deciding to stop data collection after six interviews due to the contrast and richness already prevalent in the data. The interview data was used to develop four typical case vignettes [22] of patients with dementia to be featured in focus groups (all case vignettes can be found in the Additional file 1).

The project coordinator also conducted semi-structured interviews with six leading neuroscientists at the DZNE to understand their approach to investigate dementia and how it might overlap with the experiences of GPs. The neuroscientists were asked to explain their research projects, how much contact they have with dementia patients, and how they envision working with GPs. The interviews (recorded by note taking) lasted from 30 to 45 min and took place in the researcher’s offices. The interviews were combined with observations of neuroscientists at three conferences held at the DZNE.

### Stage 2: focus groups with GPs to discuss typical case vignettes of patients with signs of dementia

Based on the interview results, the two project leaders (one is a GP) and the project coordinator constructed four case vignettes to be used in the focus groups. Two focus groups were held in stage 2 and two more in stage 3 of the project. Eight quality circles in the area were contacted and asked if they wanted to participate as a focus group. The circles that agreed to participate and had scheduled a meeting in the near future were chosen. In stage 2, two focus groups were held, one in an urban and the second in a rural setting for comparison. The focus group method was selected to gain deeper insights into GP’s perspectives through their discussions with peers about the specific cases presented. In contrast to individual interviews, focus groups have the added value of allowing discussion to bring participants’ attitudes and beliefs to the surface. Additionally, interaction among participants can be observed [23, 24].

The focus group meetings lasted 1 h and were audio recorded. Additionally, two research assistants took field notes. All participating GPs were numbered according to their seat at the table. When a GP spoke his number plus the first sentence was written down to differentiate the participants in the recording and transcript afterwards. The moderator introduced briefly the research, explained the consent form and answered questions from participants. Then the four case vignettes were introduced and discussed sequentially. The data was analyzed similarly to the individual interviews using the software MAXQDA as an organizing tool again.

The four case vignettes described the following situations: a) a single man at the age of 65 who showed signs of dementia, b) a wife (age 77) that recognizes signs of dementia in her husband (age 82) and asks her GP for help, c) a wife (age 83) who has difficulties taking care of her husband (age 88) with dementia and refuses help, and d) a patient (age 50) who believes he shows signs of dementia. The case vignettes exemplify what GPs experience in their daily practices (all case vignettes can be found in the Additional file 1).

The discussion among the GPs revealed the extent to which the case vignettes reflected their experiences and showed their strategies for managing dementia in dealing with these situations. The focus of conversation among the GPs included specific approaches of establishing a differential diagnosis and subsequent treatment, in addition, issues regarding health policies, networks of care and contacts with specialists were part of the discussion and added to the broader understanding of their actions.

We anticipated that not all GPs would contribute to the focus group discussions. Some just listened. To ensure that answers of all GPs were collected the participants were asked to fill out a questionnaire at the end of the session. The questionnaire was composed of open- and closed-ended questions, such as total number of patients during a 3 months period, most prescribed medications for dementia, use of guidelines to follow diagnostic and treatment options, experience with medication, access to a network of medical and health care specialists, interest in research with neuroscientists, and further training about dementia treatment options.

### Stage 3: focus groups with GPs to develop an observation sheet (OS) for signs of cognitive changes

Before beginning stage 3, the DZNE neuroscientists invited all GPs in Saxony-Anhalt to a professional exchange about dementia research. The NEUROTRANS-Project team presented results of stages 1 and 2 and presented six potential areas of collaborative research for GPs and neuroscientists. The GPs and neuroscientists reached consensus on one topic: to develop an observation sheet (OS) for identifying cognitive changes among patients over time. The observation sheet would include the characteristics that the GPs developed as part of the study. The OS is meant for GPs to collect as a follow-up specific information that in combination with the individual situation of patients might help to better determine when more testing is needed [25].

To develop the OS, two more focus groups were conducted (again one in an urban and one in a rural setting). Participants received a partially constructed questionnaire comprised of the most common dementia-associated behavioral changes that GPs identified in the NEUROTRANS-Project stages 1 and 2. These were used to trigger discussions to develop a more specific questionnaire that could be used during every office visit with patients in the targeted age group. As in stage 2 at the end of the focus group sessions all participants completed the questionnaire to ensure the inclusion of GPs who did not actively participate in the discussion.

## Results

The result section is organized according to the stages of the study. The individual interviews and focus group sessions with GPs present similar experience with dementia patients regardless of urban or rural location. GPs in rural areas have more problems finding colleagues and other health care professionals to work with. Both groups work in different systems (health care and research) that provide no opportunity for exchange.

### Stage 1: individual interviews and first focus group discussions with GPs; interviews with neuroscientists and conference observations

The individual interviews focused on the question of how GPs recognize symptoms of dementia in their patients, how they pursue the diagnoses, the treatment options they know and the support they seek among their colleagues.

General practitioners reported that they first suspect possible cognitive problems when they notice social or behavioral changes. Patients may wear stained clothes, look disheveled, or have body odor, suggesting difficulty in maintaining hygiene. The elderly may not be able to get to their habitual hair appointments contributing to disinterest in maintaining their appearance. Others may be quieter than usual or withdrawn, offering excuses for missed doctor’s appointments.

Rather than thinking of these as signs for dementia, however, GPs tend to consider them as stemming from other common conditions such as depression, diabetes, hearing or visual impairment, or social problems (e.g. death of a family member). As one GP noted: “When a patient shows symptoms of cognitive problems, I’ll first check organic issues -- the whole range of diagnostics. If the results don’t show a somatic cause, then I’ll check for dementia” (GP 1).

Relatives play an important role as a source of information for GPs about the possible onset of dementia. More often than the patients themselves, relatives mention inexplicable changes they see in their loved ones. GPs describe relatives’ comments as eye opening because their own observations suddenly make more sense. They also explain that consultations with patients are typically too short for them to detect signs of dementia immediately.

When GPs do suspect possible dementia, their diagnostic tools of choice are the clock test, the money counting test, and the Mini-Mental-Status-Examination. These are validated tests used to detect cognitive disorders in general practice [26]. Some of the GPs questioned the tests as being not sensitive enough to assess a beginning memory decline and not helpful in evaluating whether a prescribed medication works or not. Health insurance regulations require GPs to evaluate patients taking dementia drugs every 6 months. The GPs acknowledged that they almost see no difference in the tests.

“The thing is I am asking myself: what do I do now? I have to tell the patient the result [of the dementia test], but then what? I don’t have much I can offer. The medication might work for up to two years, and we have to check [it] every six months. But it is difficult to tell if the patient really benefits” (GP 6).

Test uncertainties are related to the issue of being able to get support from colleagues who can better diagnose dementia, such as neurologists or psychiatrists to suggest possible treatment options. “I am missing neurologists who can more quickly and better diagnose dementia than I can. Patients are supposed to make their own appointments (with neurologists). I would like to call a colleague to discuss my observations and to find out what the patient is still able to do and not to” (GP 5).

A dementia network may be loosely defined as colleagues (neurologists/psychiatrists) who can help to establish a diagnosis and treatment plan. While GPs in urban areas had contacts with neurologists GPs in rural settings had no access to a dementia network and none of them knew about the memory clinic, diagnostic options and treatment advice at the DZNE. Thus, GPs are trapped in a cycle of disregarding early memory changes because of the shame and stigma connected with the disease and some GPs question the usefulness of early diagnosis because they are unable to offer a cure.

These issues were then discussed in two focus group sessions one with seven and one with five participants. The participants were on average 52 years old (age range 38 to 68 years). They had been in practice on average 18 years (range from six to 33 years) of whom eight worked in joint offices and four in single practice. The GPs could identify with three of the four case vignettes describing: a) a single man at the age of 65 who showed signs of dementia, b) a wife (age 77) that recognizes signs of dementia in her husband (age 82) and asks her GP for help, c) a wife (age 83) who has difficulties taking care of her husband (age 88) with dementia and refuses help, and d) a patient (age 50) who believes he shows signs of dementia. Whereas case vignettes (a) to (c) were discussed thoroughly and the GPs added detailed experiences to the stories, they could not identify with (d) because they had not experienced such young patients with signs of dementia. Instead they called case study (a) an early case of dementia.

Overall, the GPs have seen an increase in the number of patients who show signs of dementia. Because their practices are already overwhelmed with patients who are suffering from chronic conditions such as diabetes and chronic heart and lung diseases, these are the diseases that tend to get their attention [27]. Additionally, the age of the practitioner seems to make a difference in the strategies they use to diagnose dementia, with younger GPs being better informed about different types of the disease and more interested in being involved in research. When asked how they judge their own knowledge about diagnosing and treating dementia five GPs considered their knowledge as ‘high’ whereas seven answered ‘average’. They also considered more information about treatment options, more and better support for relatives, alternative therapies, and treatment of symptoms, such as restlessness, aggressiveness and sleeplessness of dementia patients important.

### Interviews with neuroscientists and conference observations

In contrast to the GPs, the neuroscientists interviewed for the NEUROTRANS-Project were removed from the social worlds and daily lives of patients with dementia. With backgrounds mostly in the fields of medicine, biology, physiology, psychology, and physics, they tend to work in interdisciplinary and international teams and have English as a common working language (GPs working language is mostly German). Those without a medical background (for example M.Sc. of Neuroscience) usually have no clinical experience with dementia patients.

Whereas GPs referenced commonly known forms of dementia (e.g., Alzheimer’s Disease, vascular dementia), the neuroscientists discussed about 50 known types of dementia and the specific tests that can be used to differentiate them. While explaining their work, they talked about bio-markers and changes in brain structure that can be viewed through imaging techniques. Although the DZNE offers an open access memory clinic, patients’ experiences are not central to their thinking. In addition, people attending the clinic are often self-selected worried well. Those “worriers” are often well educated and informed about the increase of dementia patients. They do get tested and often perform so well that they cannot be included in clinical trials, making them less useful to neuroscientists focused on research scenarios.

The neuroscientists talked about the pressure from multiple stakeholders to present impressive research results. With eyes on the (Nobel-) prize, politicians look to researchers to find a cure for Alzheimer’s disease. Practitioners and patient advocates alike hope for scientific breakthroughs that will make a real difference in combating a constellation of diseases that is devastating elder populations around the globe. In an expensive and competitive field of research, neuroscientists must constantly apply for funding to develop their projects. Doing so successfully requires a prolific publishing record in journals with high impact factors.

Neuroscientists are aware of the need for clinical applications of their work. In fact, several of the scientists noted that the tests GPs use to diagnose cognitive changes simply cannot detect dementia in its very early stages, and they would like to develop a test to recognize more subtle changes [28]. Currently, several DZNE projects focus on alterations in brain physiology that probably start 10 to 20 years before recognizable cognitive signs emerge (pre-clinical stage). Other projects use declining cognitive abilities as indicators of dementia in an early clinical stage, including the diagnosis of mild cognitive impairment (MCI) and moderate dementia. Looking to get referrals of patients with signs of dementia to recruit for their research, neuroscientists admitted that they were unsure of how to work with GPs to do so. The different languages, priorities, experiences, and perspectives on patients between the two groups create a communication barrier that can make collaboration challenging.

### Stage 3: focus groups with GPs to develop an OS for signs of cognitive deficits

A professional meeting between neuroscientists (6) and general practitioners (16) at the DZNE workshop highlighted important differences in their thinking about dementia. After the NEUROTRANS-Project team presented their research findings on the practitioner context, the neuroscientists introduced DZNE research projects they believed to have collaborative potential for researchers and GPs, followed by a discussion of dementia patients.

The main point of contention between practitioners and neuroscientists was the ethics of diagnosing dementia as early as possible to gain more insight into the development of the disease and to enhance the chance of an earlier treatment. Whereas the neuroscientists view early diagnosis as paramount to their work, practitioners are skeptical about the value of early detection without a readily available, effective treatment. Neuroscientists argue that although disease modifying treatments have failed to show anticipated results and foundational research is far from finding a cure, dementia has to be treated before symptoms become visible (possibly 10 to 15 years in advance) (see Table 1). Since GPs see their patients regularly and have known many of them for years, the neuroscientists believe that GPs will be able to detect very subtle changes, thereby reducing the risk of misdiagnosis. However, practitioners admitted openly in interviews that they did not share this same confidence as it may take years before signs of dementia emerge in the clinical setting. Table 1 presents the views of both groups regarding early detection of dementia. Whereas the neuroscientists’ argument is that the earlier they can detect types of dementia they will be able to find a treatment it is the opposite for the GPs. The latter wants to secure the quality of life of their patients and will only agree to early detection if treatment is available. The views of the GPs are in agreement with the medical guidelines and research that do not recommend early testing for dementia and outline when testing and further diagnostics are appropriate [4, 25, 29, 30].

**Table 1:** Contrasting views of Neuroscientists and GPs regarding early detection of dementia symptoms

**Neuroscientists at the professional meeting:**	**General Practitioner third stage focus group:**
Therapies that tried to modify the disease have failed. This means that when signs of dementia become visible, it is too late to treat the actual cause and [the treatment] will not be effective. We need to detect the disease as early as possible before relevant symptoms occur. We need to [be able to] say if a patient *might* develop dementia in 10 to 15 years.	I believe it doesn’t make any sense to tell a patient very early that he might have dementia because it will not have any consequences. Even if you tell me that there are medications that will delay the progress of dementia for 2 years the patient will not have two good years. With the diagnosis, I have destroyed his life. In my belief, it doesn’t make any sense to detect it (dementia) early.

Without reaching agreement on whether early diagnosis or disease management (information regarding therapy options, behavior and handling of the disease, information about help and support options and such) should be the primary goal in dementia research, GPs were generally open to participating in neuroscientific research. Corresponding with Rosemann & Szecsenyi however, few said they would participate in developing a project from start to finish primarily due to time constraints [31]. Again, younger GPs were more interested in research, and overall GPs said that they would like to know more about dementia in terms of types and treatments available. Neuroscientists also learned from the exchange, that GPs have a different view about the disease based on insights from their work that researchers do not have. The GPs freely expressed their ideas and questions instead of just listening to the presentations thereby turning the event into a knowledge exchange and circulation between equal partners.

Following the professional meeting of neuroscientists and GPs two more focus groups were conducted to develop the OS. A total of 20 GPs participated (eleven women, nine men), they were on average 55 years old (age range 41 to 70 years). They had been in practice on average 19 years (range 6 to 25 years), nine GPs in joint practice and 11 in single practice. The participants received the OS that included some of the observations from the focus groups stage 2, such as changes: in appearance (difficulties in maintaining hygiene), not attending scheduled doctor appointments, not picking up medication regularly, being quieter or withdrawn than usual and so forth. These focus group sessions showed the challenges of administering the OS in general practice. The issues discussed were not so much the content of the OS but to a greater degree of how to incorporate it into daily practice. The time constraints of the daily office routine were viewed as a substantial problem. Other issues focused on the research plan, for example how to ensure that the observational sheet can be utilized to all patients above the age of 60 (some patients come in often others only once per year or even less). Who should utilize the OS (GPs or the medical assistant)? Many offices do not have a computerized system allowing for collecting the data digitally and share it easily with the DZNE. The examples show the barriers that make it difficult to involve GPs into research and have to be taken into account while planning the research. Overall, nine out of the 20 GPs declared their interest to participate with researchers at the DZNE to test a timely OS. Five participants would refer patients to the DZNE and three GPs were interested to develop a research project with the neuroscientists from the planning stages to the end. When asked for their reasons to participate they said that the advancement of knowledge was the biggest factor.

## Discussion

The NEUROTRANS-Project shows that challenges to knowledge circulation and research collaboration between neuroscientists and general practitioners are not easily to overcome. From the outside, it looks that both groups deal with the same disease however, their different professional orientations shape their views about dementia in profound ways. What’s more, while they are uniquely prepared for their respective professional careers, they are not at all primed to work together.

While GPs interact with a patient’s social environment to establish a dementia diagnosis and determine appropriate medical treatment and care, they also hold strong views against early testing for dementia and are in accordance with professional guidelines and research [32–34]. They rely on their medical knowledge, years of experience, professional exchanges such as ‘quality circles,’ and the personal interactions they have had with patients over many years to establish trust and gain insight into their every-day-life. Our results matched GPs’ behavior in other parts of Germany [3–7, 35]. By nature of their daily professional activities, they are less likely to be involved in research projects, but there seems to be a shift that younger GPs who have finished their degrees more recently received more knowledge about research through their medical studies than the generations before them. This is important for the professional development of GPs and the further practice of EBM.

Neuroscientists have limited contact with patients other than research participants and focus their work on the molecular signs and biological pathways of different types of dementia hoping to detect it as early as possible and find the ultimate cure. Our observations suggests that physio-pathological changes in brain structure and bio-markers hold neuroscientists’ core interests, not the individual patient with dementia. At the conferences, most poster and power point presentations showed MRI pictures of activity in structures of the brain. However, three of the six neuroscientists interviewed were involved in research projects that took the social context of people with dementia somewhat into account and showed some overlap with the experiences and knowledge of the GPs. As a result, general practitioners may lack insights from research that could impact how they treat patients while neuroscientists might not consider realities of dementia patients’ lives that could broaden their research priorities.

The different perspectives, experiences, and contexts of GPs and neuroscientists are critical to understand if research collaborations with these two groups are to be successful. First, GPs and neuroscientists assign different meanings to dementia. While GPs think of the consequences’ dementia has on the lives and futures of their elderly patients, neuroscientists tend to be fixated on the brain physiology of asymptomatic people in their thirties or forties. Neuroscientific research guides, clinical protocol and treatment options, contribute to the issue that the neuroscientist as ‘knowledge producer’ are removed from the practitioner as ‘knowledge user’ and knowledge itself is not easily transferred between the two groups [36].

Second, comprehensive evidence about dementia must be taken into account to further collaborations between neuroscientists and GPs. For example, the literature supports GPs’ contention that early detection of preclinical dementia might not be beneficial for patients. Studies of mild cognitive impairment (MCI) show that the benefit of testing is small [37], that advice to modify risk factors and lifestyle changes should be good practice anyway [38], that testing for MCI can lead to the stigmatization of patients, and that three-quarters of diagnosed MCI cases do not develop into Alzheimer’s disease [4]. Neuroscientists, therefore, need to find out more about the specific group that will develop Alzheimer’s disease before (early) detection occurs - and with it, anxiety for people who might not develop it. The recommendations by ALCOVE (ALzheimer’s COoperative Valuation in Europe) concentrate on the outcomes of early testing for patients and recommend a “timely diagnosis” so that patients, family members and care givers can make decisions when they benefit the most [39]. Furthermore, terms such as, “screening” and “early diagnosis” are understood differently by various health professionals contributing to the contention in the field. A strategy for timely diagnosis should maximize benefit and minimize harm. Overall, these recommendations support the concerns voiced by the GPs in the NEUROTRANS-Project.

Third, because the primary focus for GPs is on patient care and his lifeworld based on a trusting relationship rather than disease treatment per se, joint research projects must include care pathways as well. The NEUROTRANS-Project confirms findings from other studies about GPs’ motivations and decisions to support patients and relatives when signs of dementia emerge [6, 35]. GPs are already responsible for advising patients and relatives to ensure good care. Acutely aware of the support (or lack thereof) provided by the German health care system [40], they are becoming less treatment focused and more managerial in order to create systems of care and support for their patients with dementia. Thus, the role of the health care system in GPs professional orientation needs to be evaluated.

Fourth, our finding that time constraints are one of the greatest barriers to research participation for GPs makes sense when considering the local and professional contexts in which both GPs and neuroscientists operate. As the administrative and organizational duties of GPs increase, patient contact hours automatically decrease [41]. Time is also an important factor for neuroscientists for whom the lag from R&D to market is long and cumbersome. Although we found GPs to be open to research participation, the projects most suitable for collaboration with neuroscientists may not be the kind of studies likely to get funding and R&D support.

Fifth, neuroscientists are embedded in a science system shaped by strong research paradigms (see also interviews with neuroscientists) [1, 11–13] and controlled by the research agenda of funding agencies. The funding system in Germany (mostly publicly held) supports a positivistic view of research and prioritizes publishing in high impact journals, particularly those that are internationally recognized. Most funding goes to medical foundational research rather than health services research oriented towards patients’ needs [42].

Sixth, office based neurologist do support GPs in terms of dementia diagnosis and treatment with cholinesterase inhibitors (ChEIs) or NMDA-receptor-antagonists (memantine). As a rule, however, they do not provide continuing care and meet the same obstacles in conducting patient centered research as the GPs.

Overall, the results of NEUROTRANS show that only through an active effort of collaborative practice and research GPs and neuroscientists are able to share their knowledge and experience. Knowledge circulation between GPs and neuroscientists will not occur naturally and by itself. Both groups have to be actively brought together by funding agencies, universities, clinics, and professional organizations to foster a new approach for developing research collaborations, such as “bridge projects” between GPs and neuroscientists. Since the conclusion of the NEUROTRANS-Project some of the neuroscientists of the DZNE included social perspectives in their newer projects focusing on the environment of the patients and its influence on the dementia progression. These projects focus, for example on dance routines and its cognitive impact, the use of virtual reality headsets with patients, and ergometer training with another senior.

**Fig. 2:**
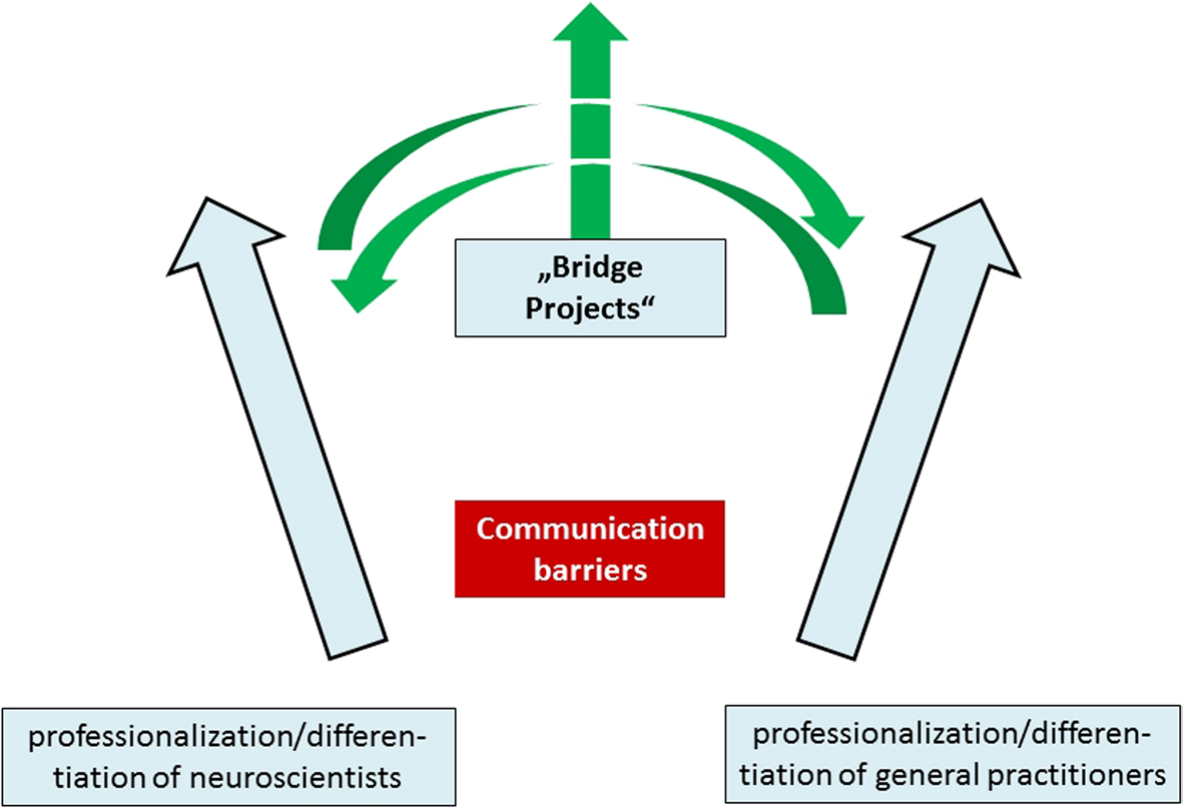
Minimizing communication barriers and supporting knowledge circulation through bridge projects

Figure 2 presents the situation of the neuroscientists and the GPs in career paths that start apart from each other and develop further in different directions without having any contact or exchange thus both groups become specialists in their fields. A “bridge” to connect both groups could be research projects that are planned ideally by members of both groups to share knowledge, expertise and responsibilities.

Bridge projects might help to overcome professional barriers between neuroscientists and GPs. Specifically, they aim to broaden the neuroscientific research perspective by involving practitioners in a research project at the early stages, thereby overcoming the evidence-to-practice gap [43]. Doing so requires the incorporation of practice into knowledge production itself: bringing the art of clinical practice into systematic scientific inquiry while also bringing scientific evidence into the art of patient care – what we call ‘art into science and science into art’. In this scenario, practitioners benefit from an increased understanding of disease progression and the different types of dementia while the knowledge GPs acquire from their daily work in a comprehensive long-term relationship with patients reflectively feeds into the production of scientific evidence [44]. Narrative medicine provides a framework for the art of patient care done by GPs [45]. Evidence-based medicine combined with narrative based medicine provides the framework for such action.

## Conclusion

Neuroscientists view foundational research as the most meaningful process whereas general practitioners focus on what benefits their patients. In order to work together both groups have to understand and include the view of the other. This is a challenge for both groups. The individual GP has to be open to reflect on his knowledge and to integrate new knowledge in the treatment of his patients. Neuroscientists must do the same thing as they develop patient-centered, evidence-based research agendas.

Knowledge circulation does not happen in a vacuum, but in order to secure the health and autonomy of the growing number of old people new ways in research and practice have to be found. Further specialization of researchers and practitioners will not lead to closing the evidence to practice gap - it will rather deepen it. The goal should be to initiate projects where both groups are able to share their knowledge, learn from each other, and produce new knowledge with each other. Strategies such as, competency networks, integrative research and treatment centers, more clinical and research learning opportunities for students in science and medicine, a funding system that supports joint projects of scientists and practitioners, regional quality circles for different health services professions, and more opportunities for health services research are useful for bringing researchers and practitioners together in order to support knowledge circulation and interdisciplinary research. The NEUROTRANS-Project is the first to suggest establishing research that builds a bridge between two groups where meaning and knowledge of both is shared and valued to work on important questions and solutions together to close the evidence to practice gap.

## Limitations

This research is exploratory with a small sample size and self-selected participants therefore it cannot be generalized to the total population of GPs in Germany. However, our data from the individual interviews, the focus group discussions and the observations is comparable to the findings of research that looked at GPs and dementia in Germany. Problems of cooperation and subject specific re-integration in a specialized division of labor in science are more general [4, 5, 7, 35, 40, 41, 46].

## Supplementary Information

**Additional file 1.**

## Data Availability

The datasets generated and analyzed during the current study are not publicly available due to protection regulations of the European Union [47], the data protection regulations of Germany and the data protection regulations of the Otto-von-Guericke-University Magdeburg, but are available from the corresponding author on reasonable request. The original data is in the German language. The data is stored for 10 years password secured electronically according to the data protection regulations of Otto-von-Guericke-University Magdeburg.

## References

[1] Vollmar HC, Santos S, de Jong A, Meyer G, Wilm S (2017). Wie gelangt Wissen in die Versorgung? Implementierungsforschung und Wissenszirkulation. Bundesgesundheitsblatt Gesundheitsforschung Gesundheitsschutz.

[2] Straus SE, Tetroe J, Graham I (2009). Defining knowledge translation. CMAJ.

[3] Kaduszkiewicz H, Röntgen I, Mossakowski K, van den Bussche H (2009). Tabu und Stigma in der Versorgung von Patienten mit Demenz: Kann ein Fortbildungsangebot für Hausärzte und ambulante Pflegedienste zur Destigmatisierung beitragen?. Z Gerontol Geriatr.

[4] Kaduszkiewicz H, Eisele M, Wiese B, Prokein J, Luppa M, Luck T (2014). Prognosis of mild cognitive impairment in general practice: results of the German AgeCoDe study. Ann Fam Med.

[5] Thyrian JR, Hoffmann W (2012). Dementia care and general physicians - a survey on prevalence, means, attitudes and recommendations. Cent Eur J Public Health.

[6] Pentzek M, Fuchs A, Abholz H-H (2005). Die Einstellungen der Hausärzte zu Demenzen. Nervenheilkunde.

[7] Pentzek M (2005). Der Mini-Mental-Status-Test (MMST) als Demenz-Screening: Methodische Überlegungen zur Eignung in der Hausarzt-Praxis. Z Allg Med.

[8] Chinthapalli K (2014). Alzheimer’s disease: still a perplexing problem. BMJ.

[9] Lau R, Stevenson F, Ong BN, Dziedzic K, Treweek S, Eldridge S (2016). Achieving change in primary care--causes of the evidence to practice gap: systematic reviews of reviews. Implement Sci.

[10] Arora S, Thornton K, Komaromy M, Kalishman S, Katzman J, Duhigg D (2014). Demonopolizing medical knowledge. Acad Med.

[11] Ioannidis JPA (2006). Evolution and translation of research findings: from bench to where?. PLoS Clin Trials.

[12] Sackett DL, Rosenberg WMC, Gray MJA, Haynes BR, Richardson SW (1996). Evidence based medicine: what it is and what it isn’t. BMJ.

[13] Bensing J (2000). Bridging the gap. The separate worlds of evidence-based medicine and patient-centered medicine. Patient Educ Couns.

[14] Aurenque D, Klitzke K. BMBF-Klausurwoche (ELSA): Würde und Autonomie als Leitprinzipien in Theorie und Praxis der humanen und außerhumanen Lebenswissenschaften. Ethische, rechtliche und theologische Dimensionen. Ethik Med. 2011. 10.1007/s00481-011-0143-y.

[15] Zwart H, Landeweerd L, van Rooij A (2014). Adapt or perish? Assessing the recent shift in the European research funding arena from ‘ELSA’ to ‘RRI’. Life Sci Soc Policy.

[16] Rohrbasser A, Mickan S, Harris J. Exploring why quality circles work in primary health care: a realist review protocol. Syst Rev. 2013. 10.1186/2046-4053-2-110. 10.1186/2046-4053-2-110 PMC4029275 24321626

[17] ÄZQ: Qualitätszirkel. https://www.aezq.de/aezq/kompendium_q-m-a/6-qualitaetszirkel/#. Accessed 27 Apr 2021.

[18] Hänel P, Lichte T, Herrmann M (2014). SIQ: Unterstützung für hausärztliche Qualitätszirkel in Sachsen-Anhalt. Z Allg Med.

[19] Kassenärztliche Vereinigung Sachsen Anhalt (Association of Statutory Health Insurance Physicians of Saxony-Anhalt): Qualitätszirkel. 2020. https://www.kvsa.de/praxis/fortbildung/qualitaetszirkel.html. Accessed 6 Apr 2021.

[20] Glaser BG, Strauss AL (1967). The discovery of grounded theory: strategies for qualitative research.

[21] Creswell JW (2013). Qualitative Inquiry & Research Design: choosing among five approaches.

[22] Cox K (2001). Stories as case knowledge: case knowledge as stories. Med Educ.

[23] Scott G, Garner R, Lewis-Elligan T. Focus groups. In: Scott G, Garner R, editors. Doing qualitative research: designs, methods and techniques. New Jersey: Pearson Education Inc; 2013. p. 298–310.

[24] Freitas H, Oliveira M, Jenkins M, Popjoy O. The focus group, a qualitative research method. Reviewing the theory, and providing guidelines to its planning, ISRC Working Paper 010298; 1998. http://gianti.ea.ufrgs.br/files/artigos/1998/1998_079_ISRC.pdf. Accessed 6 Apr 2021.

[25] S3-Leitlinie: Demenzen. https://www.awmf.org/uploads/tx_szleitlinien/038-013l_S3-Demenzen-2016-07.pdf. Accesses 25 June 2021.

[26] MDK Kompetenz-Centrum Geriatrie: Assessments in der Geriatrie. 2020. http://kcgeriatrie.de/Assessments_in_der_Geriatrie/Seiten/default.aspx. Accessed 6 Apr 2021.

[27] Arvidsson E, André M, Borgquist L, Carlsson P (2010). Priority setting in primary health care - dilemmas and opportunities: a focus group study. BMC Fam Pract.

[28] Berron D, Düzel E, Jessen F, Bug C (2019). Digitales Monitoring von spezifischen kognitiven Beeinträchtigungen in der frühen Alzheimer-Erkrankung. Disease interception.

[29] Owens DK, Davidson KW, Krist AH, Barry MJ, Cabana M, Caughey AB (2020). Screening for cognitive impairment in older adults: US preventive services task force recommendation statement. JAMA.

[30] Patnode CD, Perdue LA, Rossom RC, Rushkin MC, Redmond N, Thomas RG (2020). Screening for cognitive impairment in older adults: updated evidence report and systematic review for the US preventive services task force. JAMA.

[31] Rosemann T, Szecsenyi J (2004). General practitioners’ attitudes towards research in primary care: qualitative results of a cross sectional study. BMC Fam Pract.

[32] NHS. Dementia guide. 2020. https://www.nhs.uk/conditions/dementia/early-diagnosis-benefits/. Accessed 6 Apr 2021.

[33] Fox C, Lafortune L, Boustani M, Brayne C (2013). The pros and cons of early diagnosis in dementia. Br J Gen Pract.

[34] Watson R, Bryant J, Sanson-Fisher R, Mansfield E, Evans T-J. What is a ‘timely’ diagnosis? Exploring the preferences of Australian health service consumers regarding when a diagnosis of dementia should be disclosed. BMC Health Serv Res. 2018. 10.1186/s12913-018-3409-y. 10.1186/s12913-018-3409-y PMC6080387 30081889

[35] Boise L, Camicioli R, Morgan DL, Rose JH, Congleton L (1999). Diagnosing dementia: perspectives of primary care physicians. Gerontologist.

[36] Carlile PR (2004). Transferring, translating, and transforming: an integrative framework for managing knowledge across boundaries. Organ Sci.

[37] Lin JS, O’Connor E, Rossom RC, Perdue LA, Eckstrom E (2013). Screening for cognitive impairment in older adults: a systematic review for the U.S. preventive task force. Ann Intern Med.

[38] Le Couteur DG, Doust J, Creasey H, Brayne C (2013). Political drive to screen for pre-dementia: not evidence based and ignores the harms of diagnosis. BMJ.

[39] Brooker D, La Fontaine J, Evans S, Bray J, Saad K (2014). Public health guidance to facilitate timely diagnosis of dementia: Alzheimer’s Cooperative Valuation in Europe recommendations. Int J Geriatr Psychiatry.

[40] Roos M, Krug D, Pfisterer D, Joos S (2013). Professionalität in der Allgemeinmedizin in Deutschland – eine qualitative Studie zur Annäherung an das Kompetenzfeld. Z Evid Fortbild Qual Gesundhwes.

[41] Herrmann M, Lehmann B, Dick M, Marotzki W, Mieg H (2016). Die Allgemeinmedizin. Handbuch Professionsentwicklung.

[42] Bundesministerium für Forschung und Entwicklung (BMBF). Bundesbericht Forschung und Innovation 2018. Forschungs- und innovationspolitische Ziele und Maßnahmen. BMBF; 2018. https://www.bmbf.de/upload_filestore/pub/Bufi_2018_Hauptband.pdf. Accessed 6 Apr 2021.

[43] Robra BP, Dick M, Marotzki W, Mieg H (2016). Evidenzsicherung in der medizinischen Praxis. Handbuch Professionsentwicklung.

[44] Robra BP, Dick M, Marotzki W, Mieg H (2016). Evidenz. Handbuch Professionsentwicklung.

[45] Greenhalgh T. Narrative based medicine: narrative based medicine in an evidence based world. BMJ. 1999. 10.1136/bmj.318.7179.323. 10.1136/bmj.318.7179.323 PMC1114786 9924065

[46] Felt U, Nowotny H, Taschwer K (1995). Wissenschaftsforschung: Eine Einführung.

[47] European Parliament (2016). Regulation (EU) 2016/679.

