# Supplementary material for: Finding common (research) ground between general practitioners and neuroscientists: the vital role of knowledge circulation in closing the evidence-to-practice gap
Source: BMC Fam Pract. 2021 Oct 20;22:211. doi: 10.1186/s12875-021-01560-3 (PMC8527800; doi:10.1186/s12875-021-01560-3)
Supplement: Supplementary file 1 — Additional file 1. [file 12875_2021_1560_MOESM1_ESM.docx]

Case Vignettes of the NEUROTRANS-Project Manuscript: “Finding common (research) ground between general practitioners and neuroscientists: the vital role of knowledge circulation in closing the evidence-to-practice gap”

| Mr. Smith is a 65-year-old well-groomed man who has been a patient of yours for 15 years. He lives alone and has no relatives living close to him. He participates in all preventive check-ups and is cooperative. He is slightly overweight and has high blood pressure. He always picks up his medication prescription on time. You realize that he is not doing that for the last couple of months. The office nurse contacted Mr. Smith at home and he explained haphazardly that something else came up. He looks disheveled when he comes into your office the next day. When you ask him about his children and grandchildren in Munich he answers elusive and quite in contrast to his usual communicative self. | Mr. and Ms. Delany have been your patients for many years. Mr. Delany is 88 and Ms. Delany 83 years old. Ms. Delany has arthritis in her knees and high blood pressure. Mr. Delany has dementia diagnosed with the clock-test and the MMST receiving 10 out of 30 points. For two years, you have been treading him with anti-dementia medication. You keep him on a regular office visit schedule to observe changes and the effectiveness of the medication. Mr. Delany relies heavily on his wife and you realize that Ms. Delany looks exhausted. You have the opportunity to talk with Ms. Delany alone while Mr. Delany gets an EKG. Ms. Delany explains that Mr. Delany’s day-and-night routine is dysfunctional and she does not get any quiet time for herself. |
| --- | --- |
| Questions for the focus groups:   1. Is the case study understandable? 2. What problems do you detect? 3. Is there information missing? 4. What would you do? | Questions for the focus groups:   1. Is the case study understandable? 2. What problems do you detect? 3. Is there information missing? 4. How can you help Ms. Delany? |

| Ms. Rendel is 77 years old and lives together with her 82-year-old husband. For many years, both have been patients of yours. Ms. Rendel says that she does not know what to do with her husband; he is very forgetful but does not acknowledge it. He was an executive in a large business and did not want to go into retirement. Since his retirement, he does the shopping and takes care of the household finances. Lately, he does not buy everything on the shopping list instead he buys things that were not on the list and it takes a long time for him to return home. When she cleaned his desk at home, she found numerous bills and repeated reminders from different companies. She is very concerned about her husband and asks you for help. | Mr. Meyer is 50 years old and moved recently to the area. He visits your office because he wants a flu vaccination. He tells you that he lives down the street and choose your practice because you are taking new patients. He gets his vaccination and leaves the office. After a couple weeks, he returns and wants to talk with you. He moved with his wife for the job to the area because he was tired of spending 2 to 3 hours in the car every day. For some weeks, he recognizes that he has become very forgetful and is concerned about it. He needs to write down everything otherwise it will be forgotten. He is a product manager and his work is challenging and interesting. His wife believes that he has too much stress but he does not. |
| --- | --- |
| Questions for the focus groups:   1. Is the case study understandable? 2. What problems do you detect? 3. What would you do? 4. What help can you offer Ms. Rendel? | Questions for the focus groups:   1. Is the case study understandable? 2. What problems do you detect? 3. What would you do? 4. How can you help Mr. Meyer? |
